# Logo Effects on Brand Extension Evaluations from the Electrophysiological Perspective

**DOI:** 10.3389/fnins.2017.00113

**Published:** 2017-03-08

**Authors:** Qian Shang, Guanxiong Pei, Shenyi Dai, Xiaoyi Wang

**Affiliations:** ^1^Management School, Hangzhou Dianzi UniversityHangzhou, China; ^2^School of Management, Zhejiang UniversityHangzhou, China; ^3^College of Economics and Management, China Jiliang UniversityHangzhou, China

**Keywords:** brand name, brand logo, dissimilar brand extension, N2, P300, neuromanagement

## Abstract

Brand extension typically has two strategies: brand name extension (BN) and brand logo extension (BL). The current study explored which strategy (BN or BL) better enhanced the success of dissimilar brand extension and product promotion in enterprises. Event-related potentials (ERPs) were used to investigate electrophysiological processes when subjects evaluated their acceptance of the brand extension using a combined picture of S1 and S2. S1 was a famous brand presented by two identity signs (brand name and brand logo). S2 was a picture of an extension product that belonged to a dissimilar product category than S1. The behavior data showed that BL was more acceptable than BN in the dissimilar brand extension. The neurophysiology process was reflected by a less negative N2 component and a larger P300 component in the BL than in the BN. We suggested that N2 reflected a whole conflict between the brand-product combination and the long-term memory and that P300 could be regarded as the reflection of the categorization process in the working memory.

## Introduction

Brand extension is the use of an established brand to launch a new product (Aaker, 1990; Völckner and Sattler, 2007), which serves as a critical and widespread product promotion strategy in the enterprise (Hem et al., 2003; Völckner and Sattler, 2007). A great deal of existing evidence supported the notion that brand extension obtained a higher acceptance rate when the categories of the parent brands and extension products were similar (similar brand extension) than when they were dissimilar (dissimilar brand extension; e.g., Ma et al., 2008, 2010; Jin et al., 2015). However, the dissimilar brand extension strategy also plays an important role in entering new markets for enterprises. Thus, enhancing the success of dissimilar brand extension remains a critical issue worth studying.

Aaker and Keller (1990) constructed a theoretical framework (the consumer evaluation model of brand extensions) to research the factors that influenced brand extension success. This model showed that the success of brand extension depended on the consumer's perception of how well the extension products matched the parent brand (Aaker and Keller, 1990). This finding meant that a higher perceived fit was related to a more positive evaluation of the brand extension (MacInnis and Nakamoto, 1990; Boush and Loken, 1991; Bhat and Reddy, 2001). Based on this model, the overwhelming majority of brand extension studies have focused on the perceived fit between the names of the parent brands and the extension products (e.g., Ma et al., 2008, 2014b; Wang et al., 2012; Jin et al., 2015). For example, a brand extension study by Ma et al. (2008) indicated that a higher perceived similarity and coherence between the brand name and the product name resulted in higher brand extension success. Ma et al. (2014b) suggested that a two-stage categorization process (early low-level and similarity-based processing and late analytic and category-based processing) was involved in the evaluation process of perceived fit between the names of the parent brands and the extension products (Ma et al., 2014b). However, a parent brand can primarily be identified by not only its brand name but also its brand logo (Fombrun and Van Riel, 1997; Klink, 2003; Guzmán et al., 2012). Although brand extension appears to have two important extension strategies (brand name extension and brand logo extension), few studies concerning the strategy of brand logo extension have been performed to date. Thus, it is necessary to conduct a study to examine which brand extension strategy (brand name extension or brand logo extension) is better for improving a consumer's perceived fit and enhancing the success of dissimilar brand extension.

Generally, the brand name is more simple and familiar information to consumers and is better stored in their long-term memory than the brand logo (Baxter et al., 2015). However, some studies demonstrated that the stereotypes of customers toward familiar brand information in their long-term memory could lead them to a better fit with the original product category but a worse fit with other product categories (Jin et al., 2015). In contrast, for unfamiliar brand information not stored in a consumer's long-term memory, the consumer's perceived fit between the brand information and the original product category was the same as that between the brand information and a dissimilar product category (Jin et al., 2015). Therefore, we hypothesized that the brand logo was more suitable than the brand name when a brand was extended to a dissimilar category product. Thus, the brand logo extension strategy was better compared to the brand name extension strategy in improving a consumer's perceived fit and enhancing the success of dissimilar brand extension.

To investigate how different evaluations on the brand logo extension and the brand name extension were implemented in the brain, we measured event-related potentials (ERPs) using physical picture stimuli (i.e., brand-product picture combination). ERPs are important measures of perceptual and cognitive processing of stimuli and have a high temporal resolution (Luck et al., 2000). This approach could help investigate the whole time course of the consumer's brand extension evaluation process.

N2 is an event-related potential with a negative wave peaking between 200 and 400 ms post-stimulus (Folstein and Van Petten, 2008; Dickter and Bartholow, 2010). A series of ERPs studies suggested that N2 reflected conflict and mismatch from a visual template (Van Veen and Carter, 2002; Folstein and Van Petten, 2008). For example, the N2 component has been found to have a larger amplitude when the second stimulus (S2) in a pair does not match the physical attributes of the first stimulus (S1), such as color (Semlitsch et al., 1986; Cui et al., 2000; Wang et al., 2004; Han et al., 2015), shape (Cui et al., 2000; Zhang et al., 2001; Wang et al., 2004; Han et al., 2015), orientation (Wang et al., 1998), position (Yang and Wang, 2002; Mao and Wang, 2008), or digit value (Kong et al., 2000). In these studies, the information from the S1 was first encoded into the working memory system. When the information from S2 was transmitted into the brain, the memory information from S1 was retrieved and compared with the information from S2. The difference between S2 and S1 led to memory conflict and elicited the N2 component (Han et al., 2015). In addition to the conflict between these physical attributes, perception conflict could also evoke the N2 component. For example, Ma et al. (2007, 2010) observed a greater N2 amplitude when participants perceived stronger conflict between the brand (S1) and the extension product (S2) in brand extension evaluations. The authors suggested that this perceived conflict effect resulted from the comparison of the product (S2) attribute to the brand's (S1) product attribute in the brand memory (Ma et al., 2007). Thus, it is reasonable to hypothesize that the N2 component may index as an automatic detection of memory conflict for the stimulus materials. In the current study, we hypothesized that a N2 component would be elicited by the memory conflict when the brand was extended to a dissimilar category product. Furthermore, if the brand logo extension led to a higher perceived fit compared to the brand name extension, we hypothesized that this higher fit could be reflected by a smaller memory conflict and N2 amplitude in the brand logo extension than in the brand name extension.

In addition to N2, P300 represents different aspects of the stimulus evaluation (Yeung and Sanfey, 2004; Xu et al., 2011). P300 is a positive ERPs component with a peak latency between 300 and 1,000 ms after the stimulus onset that reflects the activity of event categorization in the working memory (Kok, 2001; Zhang et al., 2003; Azizian et al., 2006; Ma et al., 2008). In a probe-matching experiment by Zhang et al. (2003), a prominent P300 was elicited when the pictures in the probe set were congruent with those in the memory set. A target-detection task experiment by Azizian et al. (2006) demonstrated that stimuli that were perceptually similar to the targets produced larger P300 responses than other stimuli. Furthermore, a recent study examined the neurophysiological process of brand extension with a prime-probe paradigm and found that a higher similarity and fit between the parent brand in the prime and the extension product in the probe resulted in a larger P300 amplitude (Ma et al., 2008). Thus, we hypothesized that if the brand logo extension could lead to a higher perceived fit than the brand name extension, then a larger P300 could be observed in the brand logo extension condition.

In the present experiment, we applied ERPs to investigate the neurophysiological process of the brand extension evaluation with two extension strategies (brand name extension and brand logo extension). The participants were presented combination pictures of the parent brand (name or logo) and the extension product. The evaluation of brand extension was measured by the subjects' acceptance (e.g., accept or not) according to previous works (Ma et al., 2008, 2014a). This study allowed us to explore which extension strategy (brand name extension or brand logo extension) enhanced the success of dissimilar brand extension and to deeply investigate the neurophysiological process underlying the brand extension evaluation.

## Methods

### Participants

Sixteen right-handed students (nine males) aged 19–23 years (mean age = 21 years, *SD* = 2.12) from Zhejiang University participated in this experiment as paid volunteers. The students were all native Chinese speakers and had normal or corrected-to-normal vision. No participants reported a history of neurological disorder or mental disease. This study was approved by the institutional ethics committee of the Zhejiang University Neuromanagement lab. Written informed consent was obtained from all participants before the experiment was formally started. Data from one subject were discarded due to excessive recording artifacts, resulting in 15 valid subjects for the final data analysis.

### Experimental stimuli

In this experiment, the target stimuli were visual pictures of extension products with a parent brand. The size of each picture was 300 × 400 pixels. The brands consisted of five categories: beverage, food, clothing, vehicle, and technology (two brands per category). All brands were selected from the “Well-known Trademark List” published by the State Trademark Administration, China. The participants were all familiar with these brands, including Coca-Cola, Pepsi, and Nike, because they were selected in advance using a special Brand Familiarity Test. The brand was separately combined with the extension product using two brand identity signs (brand name and brand logo). The extension products comprised 20 products that belonged to different categories than the original product category of the parent brand.

### Experimental procedure

The subjects were comfortably seated in a dimly lit, sound attenuated, and electrically shielded room. The stimuli were presented centrally on a computer screen at a distance of 100 cm in front of the participant. A keypad was provided for the subjects to make their choices. The experiment consisted of 2 blocks, each containing 40 trials and lasting for about 3 min. During the experiment, the subjects were presented with 40 brand name extension tasks (BN) and 40 brand logo extension tasks (BL).

**Figure 1:**
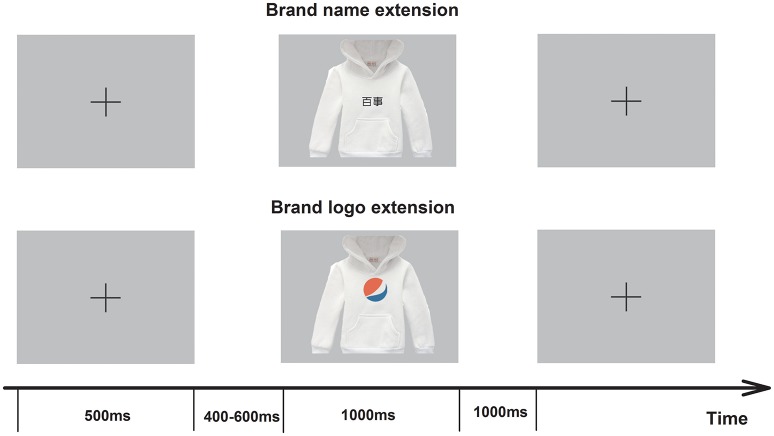
**Experimental task**. The participants were presented with two strategies of brand extension (brand logo extension and brand name extension). They were instructed to accomplish the brand extension evaluation tasks and had a maximum of 2,000 ms to make their choice. EEGs were recorded from the subjects throughout the experiment.

A stimulus system (Stim2, Neurosoft Labs, Inc., Sterling, VA, USA) was used to control the presentation of the stimuli. As illustrated in Figure 1, at the beginning of each trial a fixation appeared as a cue for 500 ms on the black screen, which was followed by an evaluation task to be performed. The evaluation task was presented for 1,000 ms and could be either a brand name extension evaluation or a brand logo extension evaluation. These evaluation tasks were randomized by the program, which made it impossible for the subjects to predict the type of upcoming task. During each evaluation task, the subjects were required to evaluate whether they would accept the presented product under the presented brand if it was sold in the marketplace. The subjects had a maximum of 2,000 ms to give their response by a button press. The response-to-hand assignments were counterbalanced across individuals. Each participant performed 10 practice trials before the start of the formal experiment.

### EEG recording

The electroencephalogram (EEG) was recorded (band-pass 0.05–100 Hz, sampling rate 500 Hz) from a set of 64 Ag/AgCl electrodes according to the 10–20 system with the Neuroscan Synamp2 Amplifier (Scan 4.3.1, Neurosoft Labs, Inc. Virginia, USA). The EEG electrodes were on-line referenced to the average of the left mastoid and later off-line referenced to the average of two mastoids. An electrode was applied to the cephalic location as the ground. Vertical electrooculograms (EOG) were recorded with one pair of electrodes placed on the supra-orbital and infra-orbital locations of the left eye, whereas the horizontal electrooculogram was recorded from electrodes on the outer canthi of both eyes. Electrode impedances were maintained below 5 kΩ throughout the experiment.

### Data analysis

For the analysis of the behavioral data, a paired *t*-test was adopted to compare the acceptance rates (AR) between the two brand extension conditions. The acceptance rate referred to the rate of like evaluations reported by the participants.

Ocular artifacts were removed during the offline EEG analysis. The EEG data were extracted from −200 to 800 ms time-locked to the onset of the task stimulus, with the pre-stimulus period used as the baseline. Electrooculogram artifacts were corrected using the method proposed by Semlitsch et al. (1986). Trials with peak-to-peak deflections exceeding ±80 μV and other artifacts were excluded. More than 35 sweeps for each condition remained, which are adequate to achieve stable and reliable measurements of N2 and P300 (Luck, 2005). The ERPs were averaged for every participant in both conditions (BN and BL). The averaged ERPs were digitally filtered through a zero phase shift (low pass at 30 Hz, 24 dB/octave).

According to previous studies on brand extension (Ma et al., 2007, 2008; Wang et al., 2012) and the scalp topographic distribution, nine electrode sites (F3, Fz, F4, C3, Cz, C4, P3, Pz, and P4) were selected for the data analysis. We averaged the ERPs amplitude of the 200–350 ms time window for the N2 component and the 400–600 ms time window for the P300 component. To study the neurophysiological features of the evaluation process on different brand extension strategies, a 2 (extension strategy) × 9 (electrode) within-subjects repeated measure ANOVA was conducted for the N2 and P300 components. The Greenhouse–Geisser (Greenhouse and Geisser, 1959) correction was applied when necessary, and the Bonferroni correction was used for multiple paired comparisons.

## Results

### Behavior results

**Figure 2:**
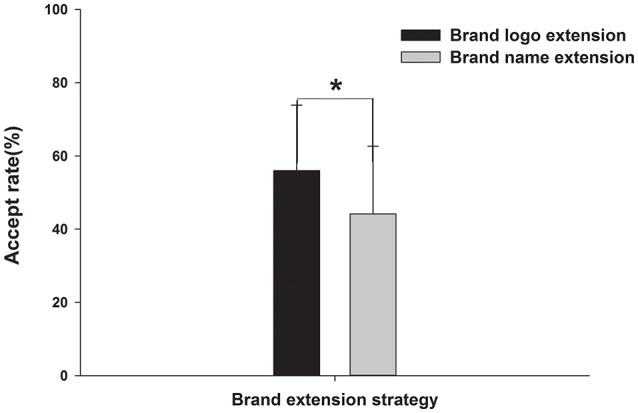
**Behavior results**. Acceptance rates of the brand logo extension (BL) and brand name extension (BN) strategies. ^*^p < 0.05.

The AR was 59.17% (SE = 3.41%) in the BL condition and 45.67% (SE = 4.47%) in the BN condition, which demonstrated a significant condition effect on the extension strategy [*t*_(14)_ = 2.194, *p* < 0.05; see Figure 2]. However, the response time in the BL condition was not significantly different with that in the BN condition [*t*_(14)_ = −1.264, *p* > 0.05].

### ERPs results

**Figure 3:**
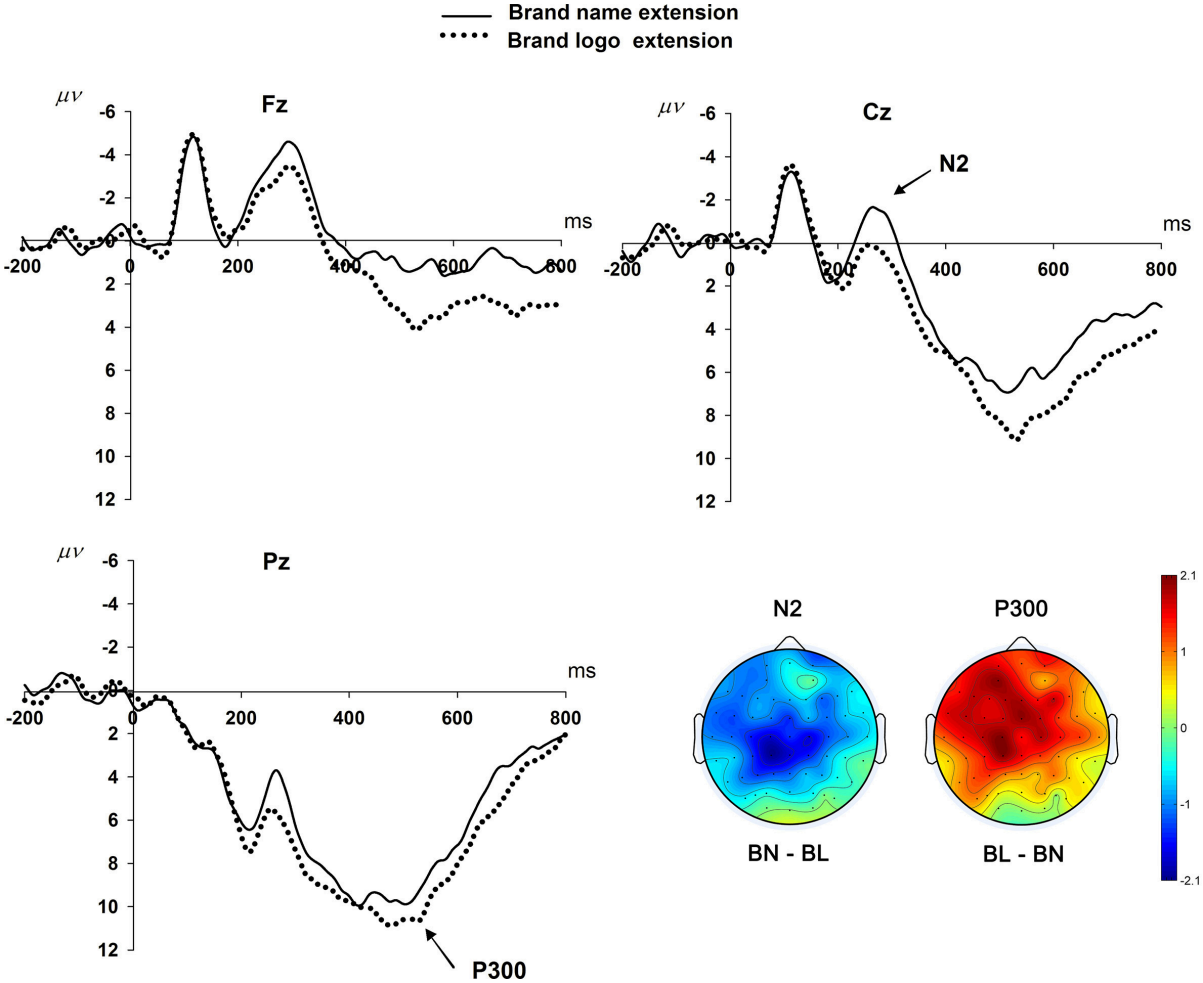
**ERPs results**. Grand averaged ERPs of N2 and P300 elicited by the extension strategies (brand logo extension vs. brand name extension) from 3 midline electrodes in the forehead, central and parietal areas (Fz, Cz, and Pz). The scalp topographic distributions of the N2 (BN condition minus BL condition) and P300 (BL condition minus BN condition) are provided, and the bar for the topographic map ranges from −2.1 to 2.1 μV.

As presented in Figure 3, the ANOVA results for N2 showed main effects of extension strategy [*F*_(1, 14)_ = 24.521, *p* < 0.001, η^2^ = 0.637] and electrode [*F*_(8, 112)_ = 26.986, *p* < 0.001, η^2^ = 0.658]. The N2 amplitude elicited by the BN condition (*M* = 1.66 μV, SE = 0.96) was more negative than the N2 amplitude elicited by the BL condition (*M* = 2.8 μV, SE = 0.97). There was no significant interaction effect between the extension strategy and electrode [*F*_(8, 112)_ = 0.541, *p* > 0.05, η^2^ = 0.037].

For the P300 competent, the ANOVA produced significant main effects of the extension strategy [*F*_(1, 14)_ = 7.198, *p* < 0.05, η^2^ = 0.356] and electrode [*F*_(8, 112)_ = 11.420, *p* < 0.01, η^2^ = 0.468]. The mean P300 amplitude in the BL condition (*M* = 6.80 μV, SE = 1.26) was significantly larger than the mean P300 amplitude in the BN condition (*M* = 5.25 μV, SE = 1.25). The interaction effect was not significant [*F*_(8, 112)_ = 0.786, *p* > 0.05, η^2^ = 0.057].

## Discussion

In the present study, we investigated how a consumer's perceived fit was influenced by different brand extension strategies (BN and BL) in the dissimilar brand extension evaluation process. Both the behavior and ERPs results demonstrated that the BL strategy could improve the perceived fit between parent brands and extension products compared to the BN strategy, which is the key to the success of brand extension. Regarding the subjects' behavior, we observed prominently higher acceptance under the BL strategy than under the BN strategy. During the neurophysiological process, a smaller N2 component and a larger P300 response were found in the BL strategy than in the BN strategy. Generally, we observed that extension products with parent brand logos showed a better perceived fit and were more favorable in the dissimilar brand extension when the subjects were shown evaluation tasks with different extension strategies.

A remarkable AR effect was found for the brand extension strategies (i.e., people showed a higher acceptance of the BL strategy than the BN strategy in the dissimilar brand extension evaluation process). As described in the Introduction, people tended to have a stereotype toward familiar brand information in their long-term memory that could lead to a better fit with the original product category but a worse fit with dissimilar product categories (Jin et al., 2015). In contrast, people's perceived fit between the unfamiliar brand information and the original product category was the same as the perceived fit between the unfamiliar brand information and the dissimilar product category because this unfamiliar information was not stored in the consumer's long-term memory (Jin et al., 2015). In the current study, brand names were more familiar information to consumers than brand logos and were stored in their long-term memory. As a result, brand names demonstrated a worse perceived fit with a dissimilar product category than brand logos. Thus, the BL strategy led to a higher perceived fit than the BN strategy. The higher perceived fit was related to a more positive extension evaluation (MacInnis and Nakamoto, 1990; Boush and Loken, 1991); hence, the BL strategy enhanced the acceptance of a dissimilar brand extension.

Regarding ERPs components, the N2 component reflects the conflicting information process (Van Veen and Carter, 2002; Folstein and Van Petten, 2008). As elaborated in the Section Introduction, a greater N2 amplitude would be observed when a conflict existed between S2 and S1 on either the physical attributes (Wang et al., 1998, 2004; Cui et al., 2000; Kong et al., 2000; Zhang et al., 2001; Yang and Wang, 2002; Mao and Wang, 2008; Han et al., 2015) or the brand perception (Ma et al., 2007, 2010). In the current study, brand names demonstrated a worse perception fit with the dissimilar product category than brand logos. Thus, the conflict between brands and products was larger for the BN strategy than the BL strategy, which was reflected on the enlarged N2 amplitude in the BN condition. However, this conflict effect was different from that reported in previous studies, which used a S1-S2 paradigm to examine the matching tasks (Wang et al., 1998, 2004; Cui et al., 2000; Kong et al., 2000; Zhang et al., 2001; Yang and Wang, 2002; Ma et al., 2007, 2010; Mao and Wang, 2008; Han et al., 2015). In these studies, the two stimuli (S1 and S2) were presented sequentially in the experiment. The information from S1 was first and temporarily encoded into the working memory system. When the information from S2 was transmitted into the brain, the temporary memory information from S1 was retrieved and compared with the information from S2. Then, the difference between S2 and S1 led to the temporary memory conflict and elicited the N2 component. Thus, this type of conflict effect was attributed to a short-term memory conflict and could be considered a partial conflict. In contrast, in the current study, the S1 (brand) and S2 (product) stimuli were combined together into one stimulus and simultaneously presented to the subjects, which was closer to the actual marketing situation. In this case, the subjects evaluated the whole stimulus, retrieved the related information from their long-term memory, and then compared this whole stimulus with the long-term memory. The larger conflict between the two types of information led to a larger N2 amplitude. Therefore, this type of conflict effect was attributed to a long-term memory conflict, which could be considered as the whole conflict.

Following N2, a positive P300 component was found in the experiment. As elaborated in the Introduction, P300 represented the event categorization activity in the working memory, and a larger P300 amplitude would result in targets with higher similarity and coherence to the prior stimuli or to the working memory (Kok, 2001; Zhang et al., 2003; Azizian et al., 2006; Ma et al., 2008). In the current study, subjects had a stereotype toward brand name information that led to a better fit with the original product category in their long-term memory. Therefore, when the subjects were presented with a combination stimulus of a brand name and a dissimilar category product, they demonstrated a lower perceived similarity to the long-term working memory and the P300 amplitude was smaller. In contrast, people who did not have a stereotype toward brand logo information in their long-term memory exhibited a higher perceived similarity and P300 amplitude when they were presented with a combination stimulus of the brand logo and a dissimilar category product. Thus, in the present study, there was a pronounced P300 discrepancy in the evaluation process between the BL strategy and the BN strategy, indicating a higher perceived similarity between the BL strategy and the long-term working memory. This finding suggested that the extension strategy might be a significant influencing factor of perceived fit in the dissimilar brand extension evaluation process, thereby manifesting the categorization process in the working memory as reflected by the electrophysiological response of P300.

This study differed from traditional ERPs studies on brand extension in several major aspects. First, previous studies primarily used isolated word stimuli with the paradigm of S1(parent brand)-S2(extension product) to examine brand extension (Ma et al., 2007, 2008, 2010; Wang et al., 2012). For instance, in an experiment on a brand extension evaluation (Ma et al., 2007), sequential stimuli were displayed in a pair consisting of brand names (S1) and product names (S2), and a greater N270 amplitude was observed when the participants were presented with a stronger conflict between the brand category (S1) and the extension product category (S2). The recent study of Wang et al. (2012) used a similar paradigm with paired stimuli of brand names (S1)-product names (S2) but removed the conscious evaluation task on the brand extension. This approach elicited a larger N400 when the product's (S2) attributes were atypical to the brand category (S1), reflecting uncontrolled categorization processing (Wang et al., 2012). However, a drawback of these previous experiments was that the brands and the products were represented by words and appeared isolated from one another. As a result, prior to the evaluation process, the participants paid more attention to associate the two words of the stimuli together in their minds, which seriously influenced the regular evaluation process. In our current study, the target stimuli were direct physical pictures of extension products combined with the parent brand. This new paradigm and stimulus type are closer to the real marketing situation and help validly explore the brand extension evaluation process.

Second, we found that brand logo extension was another important extension strategy in addition to brand name extension and was a better strategy for dissimilar brand extension. The brand name extension strategy was primarily focused by previous researchers (e.g., Ma et al., 2008, 2010, 2014b; Wang et al., 2012; Jin et al., 2015). For example, in a brand name extension study by Ma et al. (2008), a pronounced P300 effect due to category similarity was demonstrated when subjects were required to evaluate the suitability of extending the parent brand name to a similar product category and a dissimilar product category. Ma et al. (2014b) suggested that a two-stage categorization process was involved in the evaluation of the perceived fit between the parent brand names and the extension products (Ma et al., 2014b). Jin et al. (2015) found that the association of a famous brand name with a dissimilar product category led to a worse acceptance than the strategy of new brand creation. However, in addition to this brand name extension strategy, brand logo extension is another important extension strategy because a brand can be identified by its brand logo as well as its brand name (Fombrun and Van Riel, 1997; Klink, 2003; Guzmán et al., 2012). In this study, the brand name is written in Chinese. Chinese is a special hieroglyphic language system. Chinese characters are derived from pictures representing meaning (Zhang et al., 2006). A previous ERPs study investigated the time course of brain activity for the English and Chinese characters. Chinese more quickly initiated processing of graphic form and more quickly shifted to processing of meaning than did English (Liu et al., 2003). Chinese characters are hieroglyphic or pictographic and their connections with meanings are more direct than other language systems (Lam et al., 2001; Liu et al., 2003; Zhang et al., 2006). It could be assumed that the cognitive perception of the brand name (in Chinese characters which is hieroglyphic) is not so different from that of a logo (symbol or sign which is graphical). But concerned about the brand extension, the brand logo or brand name is combined with the extension product. As the results showed, the cognitive perception of the name-product combination (BN) was so different from the logo-product combination (BL), which was reflected by a less negative N2 component and a larger P300 component in the BL than in the BN. It implied that the brand logo extension strategy would lead to an enhanced perceived fit in the dissimilar brand extension evaluation process. Thus, an additional contribution of the current study was the identification of a better strategy (i.e., brand logo extension) for dissimilar brand extension in marketing research.

## Conclusions

To conclude, the current study explored which strategy [brand logo extension (BL) or brand name extension (BN)] better enhanced the success of dissimilar brand extension. Event-related potentials (ERPs) were used to investigate the electrophysiological process when subjects evaluated their acceptance of the brand extension. We found that the BL strategy increased the acceptance and perceived fit between parent brands and extension products compared to the BN strategy. In the neurophysiology process, this effect was reflected by a less negative N2 component and a larger P300 component in BL compared to BN. We suggested that N2 reflected a whole conflict between the brand-product combination and the long-term memory and that P300 could be regarded as the reflection of the categorization process in the working memory. Generally, these findings implied that the brand logo extension strategy would lead to an enhanced perceived fit in the dissimilar brand extension evaluation process. These findings are beneficial to future marketing studies.

## Ethics statement

This study was carried out in accordance with the recommendations of Neuromanagement laboratory's ethics committee in Zhejiang University with written informed consent from all subjects. All subjects gave written informed consent in accordance with the Declaration of Helsinki. The protocol was approved by the Neuromanagement laboratory's ethics committee in Zhejiang University. All participants had normal or corrected-to-normal vision. None of them reported any history of psychiatric or neurological disorders.

## Author contributions

QS, XW, and GP conceived and designed the experiments. QS, SD, and XW performed the experiment. QS, GP, and SD analyzed the data. QS, GP, and XW wrote and refined the article.

## Funding

This work was supported by the Philosophy and Social Science Planning Project of Zhejiang Province (No. 15NDJC031YB), the Humanities and Social Sciences Foundation of the Ministry of Education of China (No. 15YJC630106 and No. 14YJC630129), the National Natural Science Foundation of China (No. 71602044 and No. 71572176), the Zhijiang Youth Social Science Fund (No. 16ZJQN030YB), the Natural Science Foundation of Zhejiang Province of China (No. LQ16G020006 and No. LQ13G010005), and the Research Center of Information Technology & Economic and Social Development of Zhejiang province.

### Conflict of interest statement

The authors declare that the research was conducted in the absence of any commercial or financial relationships that could be construed as a potential conflict of interest.
